# ELaPro, a LOINC-mapped core dataset for top laboratory procedures of eligibility screening for clinical trials

**DOI:** 10.1186/s12874-022-01611-y

**Published:** 2022-05-14

**Authors:** Ahmed Rafee, Sarah Riepenhausen, Philipp Neuhaus, Alexandra Meidt, Martin Dugas, Julian Varghese

**Affiliations:** 1 Institute of Medical Informatics, University of Münster, Münster, Germany; 2 Department of Internal Medicine (D), University Hospital of Münster, Münster, Germany; 3 Institute of Medical Informatics, Heidelberg University Hospital, Heidelberg, Germany

**Keywords:** Eligibility screening, UMLS, LOINC, Data models, Medical informatics

## Abstract

**Background:**

Screening for eligible patients continues to pose a great challenge for many clinical trials. This has led to a rapidly growing interest in standardizing computable representations of eligibility criteria (EC) in order to develop tools that leverage data from electronic health record (EHR) systems. Although laboratory procedures (LP) represent a common entity of EC that is readily available and retrievable from EHR systems, there is a lack of interoperable data models for this entity of EC. A public, specialized data model that utilizes international, widely-adopted terminology for LP, e.g. Logical Observation Identifiers Names and Codes (LOINC®), is much needed to support automated screening tools.

**Objective:**

The aim of this study is to establish a core dataset for LP most frequently requested to recruit patients for clinical trials using LOINC terminology. Employing such a core dataset could enhance the interface between study feasibility platforms and EHR systems and significantly improve automatic patient recruitment.

**Methods:**

We used a semi-automated approach to analyze 10,516 screening forms from the Medical Data Models (MDM) portal’s data repository that are pre-annotated with Unified Medical Language System (UMLS). An automated semantic analysis based on concept frequency is followed by an extensive manual expert review performed by physicians to analyze complex recruitment-relevant concepts not amenable to automatic approach.

**Results:**

Based on analysis of 138,225 EC from 10,516 screening forms, 55 laboratory procedures represented 77.87% of all UMLS laboratory concept occurrences identified in the selected EC forms. We identified 26,413 unique UMLS concepts from 118 UMLS semantic types and covered the vast majority of Medical Subject Headings (MeSH) disease domains.

**Conclusions:**

Only a small set of common LP covers the majority of laboratory concepts in screening EC forms which supports the feasibility of establishing a focused core dataset for LP. We present ELaPro, a novel, LOINC-mapped, core dataset for the most frequent 55 LP requested in screening for clinical trials. ELaPro is available in multiple machine-readable data formats like CSV, ODM and HL7 FHIR. The extensive manual curation of this large number of free-text EC as well as the combining of UMLS and LOINC terminologies distinguishes this specialized dataset from previous relevant datasets in the literature.

**Supplementary Information:**

The online version contains supplementary material available at 10.1186/s12874-022-01611-y.

## Introduction

Clinical trials are essential to advance clinical health care and evidence-based medicine [1, 2]. Efficient identification and recruitment of eligible participants is considered a key factor to the success of clinical trials [3–5] and one of its major challenges throughout the last decades [6–9]. Delayed or poor recruitment of target participants in stipulated time remains an enduring problem that leads to increased study costs and reduced power of clinical trials [4, 10–12]. Insufficient participant recruitment is one of the leading causes of early study termination and wasted research resources [13–19].

Eligibility screening is considered the cornerstone of participant recruitment and refers to applying eligibility criteria (EC) to specify the necessary characteristics of study participants who are eligible to participate in a study [20–22]. The wide adoption of Electronic Health Record (EHR) systems in recent years has resulted in large quantities of patient clinical data being available in electronic form, which led to increased interest in establishing and standardizing computable knowledge representations of EC to develop decision support tools for different research aspects e.g. matching eligible patients to clinical trials [23–25]. However, these efforts are challenged by the unstandardized free-text format of EC [26, 27]. In the last decades, different clinical terminologies have been introduced and used to encode medical concepts of EC [28]. These terminologies provided a computable form of EC despite the lack of common standards among different terminologies [21]. One of the most recognized terminology systems is the Unified Medical Language System (UMLS) [29, 30], which is considered a popular option for annotating EC because of its rich metathesaurus and interoperability with other terminologies [21, 31–35]. Over the last years, various methods and techniques have been produced and applied to extract and transform medical concepts from free text into a computable representation using encoding terminologies and annotating tools. This has enhanced the development of automated research tools that utilize patient data from repositories of EHRs to recruit patients for clinical trials [36–47].

Laboratory criteria represent one of the most common categories of EC in clinical trials [48]. There is an obvious lack of dedicated analyses and specialized data models of screening LP. A public, specialized data model in interoperable terminologies for laboratory concepts, e.g. the widely-adopted, international reference of laboratory standards named Logical Observation Identifiers Names and Codes (LOINC®) terminology, is much needed to boost computer-based decision support for automated screening for clinical trials [49, 50]**.** Ross et al. randomly selected 1000 studies from ClinicalTrials.gov and found that laboratory and diagnostic tests represent around 23% of EC in these studies [51]. In 2013 Bhattacharya et al. showed that the semantic type “Diagnostic and Lab Results” constitute the majority of inclusion criteria in both full-text and protocols of ClinicalTrials.gov [52]. Wang et al. classified laboratory and demographic EC to be among the easiest criteria to support automated queries to data repositories from EHRs [53]. Both domains possess a key advantage over other EC domains, in which they are more structured and easy to retrieve from a laboratory information system to support patient recruitment. While many core data models for demographic EC already exist, e.g. Clinical Data Acquisition Standards Harmonization (CDASH), there is a clear research gap when it comes to specialized analyses and data models for LP in eligibility screening [54].

This dataset was created by analyzing 138,225 EC extracted from 10,516 UMLS-annotated screening forms of random clinical trials registered on ClinicalTrials.gov and covering a broad range of different clinical domains (Fig. 3). The forms used in this analysis were obtained from the data repository of the Medical Data Models (MDM) portal [55, 56].

In this study, we introduce ELaPro (Eligibility Laboratory Procedures), a novel, public, LOINC-mapped, core dataset of the most frequent LP in screening for clinical trials. We use a semi-automated approach that combines an automated UMLS-based semantic analysis of laboratory concepts followed by a thorough manual expert review. The scope of this analysis is confined to LP following the definition of a “Laboratory Procedure” by The National Cancer Institute (NCI) metathesaurus [57], which is defined as **“**A medical procedure that involves testing a sample of blood, urine, or other substance from the body”. Other diagnostic procedures, e.g. radiographic or endoscopic procedures are beyond the scope of this work. ELaPro is an interoperable data model, available in multiple machine-readable formats to be utilized in developing automated screening tools that can be integrated in EHR systems to enhance the recruitment process using real-time queries applied to data repositories of EHR systems.

## Methods

### Data collection

A direct access to the local UMLS database (2021AA) as well as the Metadata Repository (MDR) [58], the main database of the MDM portal, was granted by the Institute of Medical Informatics of the University of Muenster for the purpose of this analysis. A total of 12,027 EC forms were obtained from MDR as of August 2021, of which only 10,989 were technically accessible. Out of these 10,989 EC forms, 473 non-screening EC forms (e.g. follow-up, randomization or continuation criteria) were identified and excluded from this study so that only 10,516 forms met the criteria of being screening EC forms and were therefore included in this analysis. Eligibility screening forms were identified and included in this analysis. An R-based tool was developed and used to directly access and filter EC forms of MDM portal database and connect them to their UMLS-annotated concepts, which is the “raw data of the automated semantic analysis. A list of names and DOI’s of all included EC forms on MDM portal is found in Appendix 1.

### Data analysis

#### Semantic form annotation

Typically, an EC form consists of 2 item groups; Inclusion Criteria and Exclusion Criteria. Each item group consists of items; each item represents a complete element (criterion) of inclusion or exclusion criteria. All medical concepts of each item (criterion) are coded (annotated) using UMLS codes to standardize the representation of free-text EC. The annotating process is performed by a medical expert and reviewed by a physician experienced in UMLS. The detailed process and workflow of the coding process have been thoroughly described in previous works [59–61].

#### Automated semantic analysis in R

The automated part is based on an R-based tool to facilitate extraction and analysis of UMLS codes and their semantic types from pre-annotated screening forms in MDR (*n* = 10,516) and the UMLS database. We performed an automated semantic analysis on 10,516 eligibility screening forms available on the MDM portal as of August 2021. Utilizing the structure of MDR, the developed tool was able to automatically retrieve UMLS annotations of all medical concepts within screening EC forms while excluding those from other unwanted types of EC, after that, the tool measures the frequency of occurrences (n) of these annotated concepts and sorts them according to frequency in a descending order.

**Fig. 1:**
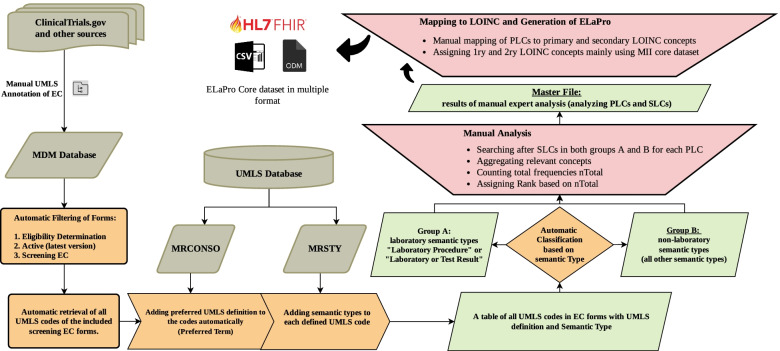
Schematic representation of the semi-automated method used in this analysis. MDR: Metadata Repository; MDM: Medical Data Models Portal; EC: Eligibility Criteria; UMLS: Unified Medical Language System; MRCONSO: a UMLS table for concept names and sources; MRSTY: a UMLS table for semantic types; SLC: Secondary Laboratory Concept; PLC: Primary Laboratory Concept; LOINC: Logical Observation Identifiers Names and Codes; MII: German Medical Informatics Initiative [63, 64]; ODM: Operational Data Model; CSV: Comma-Separated Values

In order to be able to analyze the collected UMLS codes, the developed tool used certain tables from the UMLS database to automatically assign the preferred term and semantic type of each collected code (table names are MRCONSO and MRSTY respectively [62]. Figure 1 illustrates the process of automated data collection and analysis used in this work.

In order to refine results and extract UMLS codes related to laboratory concepts, we needed to define reference semantic types that represent all laboratory concepts in UMLS metathesaurus. Based on prestudy communication with a senior scientist from the National Library of Medicine (NLM) as well as the definition of semantic types, two UMLS semantic Types, “Laboratory Procedure” and “Laboratory or Test Result”, were considered the two reference semantic types for laboratory tests in the UMLS metathesaurus.

Based on these 2 semantic types, results were divided into 2 groups; Group A was assigned the name “EC Laboratory Codes” and includes concepts (codes) from the 2 reference laboratory semantic types mentioned above, while group B was named “Non-Laboratory EC Codes” and includes codes from all other UMLS semantic types. Group B is necessary to ensure that relevant laboratory concepts, which are not linked to the aforementioned semantic types, are still considered for expert review (e.g. concepts like “Leukocytosis” or “Hemoglobin Increased” and many other concepts of semantic type “Finding”). Absolute frequency (n) was automatically counted for all codes in both groups, concepts were then sorted by absolute frequency in a descending pattern from the most frequent (highest n) to the least frequent (lowest n). Figure 1 is a schematic representation of the semi-automated method of this analysis. A list of unique UMLS concepts of group A and B sorted by frequency is found in Appendix 2A and 2B, respectively. A list of all original EC Questions for all codes in group A and B is found in Appendix 3.

#### Manual expert review of laboratory concepts

A laborious manual review was necessary to identify and analyze complex concepts that indirectly imply a LP but do not have a laboratory semantic type, thus not amenable to the above mentioned automated semantic analysis. The manual analysis was performed by 2 medical professionals (AR, JV) using Microsoft Excel. If a concept was ambiguous or in doubt it was discussed with 2 additional physicians experienced in UMLS (MD, SR) to decide whether a concept is relevant to a LP or not. We used terms like primary laboratory concept (PLC) and secondary laboratory concept (SLC) to deal with classic issues of UMLS like redundancy (similar, but not identical concepts)) and semantic complexity to help determine the actual representation (nTotal) of laboratory concepts. We provide examples for both in the following two sections.

Primary Laboratory Concept (PLC): refers to the UMLS concept that represents the preferred definition of each laboratory test in the master file. The decision of choosing the UMLS code representing each PLC was made by agreement of 4 physicians. By definition, a PLC must belong to semantic type “Laboratory Procedure” and, if applicable, be as general as possible to accommodate the different standards of the test among different clinical institutes. A PLC for a certain laboratory test is preferably, however not necessarily, the most frequent code among all codes representing that concept. For example, the concept “Creatinine Measurement in Blood” (*n* = 2) is considered the PLC for creatinine measurement despite having clearly less occurrence frequency than other more specific concepts like “Creatinine Measurement in Serum” (*n* = 1492) and “Creatinine Measurement in Plasma” (*n* = 142), since the former is more general and represents other possible variants of the test that might be used in different clinical research institutes.

Secondary Laboratory Concept (SLC): refers to UMLS concepts relevant to a PLC, i.e. it directly or indirectly refers to or implies the same laboratory test component. SLCs include concepts from laboratory semantic types (group A), that are synonymous to a PLC (sibling) as in the previous example of Creatinine, or more typically include concepts from semantic type “Finding”, which usually implies that a test is necessary to evaluate this finding, e.g. “Platelet Count Normal” or “Increased Number of Platelets” imply the need to perform the test, and are therefore secondary to the PLC “Platelet Count Measurement”. SLCs also include certain pathologic conditions that imply the need for a test, e.g. “Hyperkalemia” was considered an SLC to “Blood Potassium Measurement”, “Leukocytosis” is secondary to “White Blood Cell Count Procedure” and “Anemia” is secondary to “Hemoglobin Measurement”, etc. In some rare instances, concepts that referred to a simple relation between two measurable laboratory tests were also considered an SLC if the PLC was part of the ratio, e.g. the concept “Alanine Aminotransferase (ALT) to Aspartate Aminotransferase (AST) Ratio Measurement” was counted with both “ALT Measurement” and with “AST Measurement”.

**Fig. 2:**
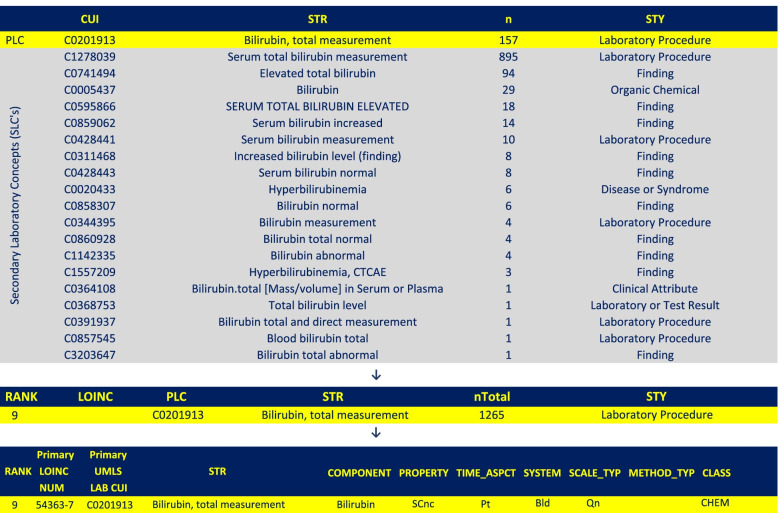
Manually analyzed concept of Bilirubin, Total Measurement. The row at the bottom shows mapping of PLC to LOINC (Further LOINC details were omitted from the image but can still be found in the dataset). PLC: Primary Laboratory Concept; STR: string (definition); n: frequency of individual concept; STY: Semantic Type; nTotal: Sum of all n’s (PLC and SLCs)

The Manual Curation (Expert Review): the most common concepts in the laboratory group A (PLCs) were identified based on the frequency of individual occurrence (n), then both A and B groups were searched to find all relevant concepts (SLCs) that directly or indirectly imply the same LP as each of the PLCs. The PLC and its SLCs are then grouped together in a master file to represent one LP (see Fig. 2). This process was repeated for each LP identified in group A. Therefore, the results of the manual analysis (master file) include multiple groups of codes, each group represents one LP and is composed of one PLC and multiple SLCs. For each LP, a total count of frequency (nTotal) was calculated by adding all concept occurrences (n) of single codes in the group representing the LP. A “Rank” was assigned to each LP based on its nTotal. The most frequent PLC (highest nTotal) was given rank number 1, second most frequent was given rank number 2 and so on. Unspecific concepts in group A (e.g. “Assay” or “Laboratory Results”) were excluded since they did not refer to any specific test component. Figure 2 shows an example of a manually analyzed LP. A diagram illustrating the manual process is in Appendix 4.

#### Mapping to LOINC®

The mapping process was based on matching the PLC to a LOINC “COMPONENT”, which is the part of LOINC that specifies what is being measured, evaluated or observed. For most LP, a primary and one or more secondary LOINC codes were assigned. The decision of choosing primary and secondary LOINC concepts was based mainly on a well-recognized core dataset created by the Medical Informatics Initiative (MI-I) that includes primary and secondary LOINC concepts for the top 300 most common laboratory tests based on data from 5 different university hospitals in Germany [63–65]. If no proper matching component was found in the MII core dataset for any of our results, the LOINC database V2.7 was directly used to manually assign a primary, and if applicable a secondary LOINC concept(s). The final step was using the UMLS database to create a core dataset with full LOINC details (component, property, system, etc.) using the LOINC codes mapped to our results and an R-based tool.

## Results

### Overview

A total of 10,516 screening forms containing a total of 138,225 criteria were recruited in this study. The MDM portal provides item group names to identify inclusion and exclusion criteria. 20,346 item groups within the 10,516 forms were identified, 9684 of these item groups (47.59%) were inclusion criteria, while 9727 (47.8%) were exclusion criteria. 932 item groups (4.59%) were unspecifically labeled.

### Representation of medical specialties among EC forms (in medical subject headings (MeSH®) terms)

**Fig. 3:**
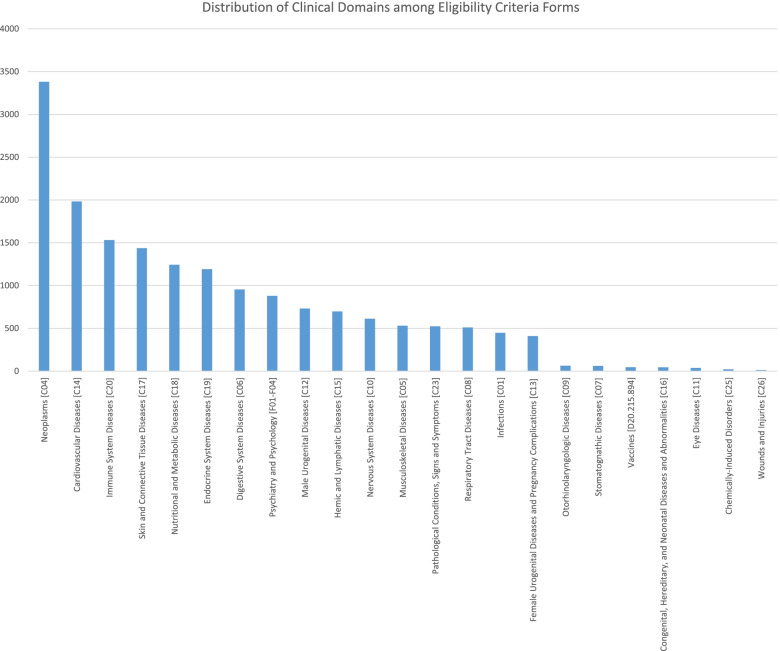
Diagrammatic representation of the distribution of clinical domains in MeSH terms among EC forms. MeSH: Medical Subject Headings

The MDM portal provides a (MeSH)-based keyword system [66]. Using this system, an automated analysis of representation of broad disease entities and medical specialties among EC forms was performed. We identified 23 unique MeSH subcategories (*n* = 17,340) among included EC forms. “Neoplasms” represent 19.49% (*n* = 3381) as the most common disease entity among EC forms. Figure 3 shows the distribution of MeSH disease entities among EC forms. Appendix 5 shows absolute frequencies of each MeSH categories.

### UMLS semantic types in screening eligibility criteria forms

A total of 27,055 unique codes were obtained from the included EC forms, among which 26,413 unique UMLS codes (97.62%) were filtered and included in this analysis. These 26,413 UMLS codes were used 495,516 times and belong to 118 unique semantic types with the most common 5 semantic types being “Finding”, “Disease or Syndrome”, “Pharmacologic Substance”, “Therapeutic or Preventive Procedure” and “Neoplastic Process” based on the frequency of occurrence of codes belonging to these semantic types (*n* = 3849, 3047, 2201, 2140 and 1204, respectively). Semantic type “Laboratory Procedure” ranked 6th and was one of the top 10 semantic types of UMLS concepts (*n* = 845), while semantic type “Laboratory or Test Result” was less frequently used (*n* = 331) and ranked 21st. Concepts from both reference laboratory semantic types combined comprised 4.45% (*n* = 1176) of all UMLS codes. Appendix 6 shows the frequency of occurrence of UMLS codes of all 118 semantic types.

### Laboratory concepts and cumulative frequencies

**Fig. 4:**
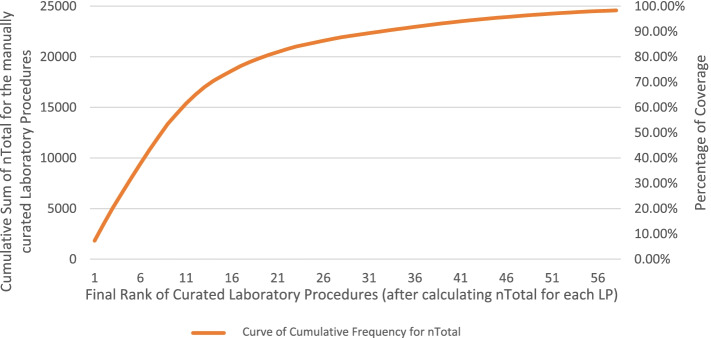
Graph showing the cumulative total frequencies among the 58 aggregated set of LP in terms of nTotal, i.e. after manual analysis and combining of all n values of PLC and SLCs for each laboratory concept. Analyzed laboratory concepts that have an nTotal above 50 concepts cover the steepest change of the slope of cumulative frequencies (Orange). Based on this, we have only included the first 55 analyzed concepts that have nTotal above 50. Blue bars show the nTotal of each analyzed laboratory concept

A total of 58 primary LP (PLCs) were identified by aggregating all relevant (secondary) concepts and calculating nTotal for each LP by adding all frequencies of individual concepts (n) of PLC and its relevant SLC. Rank was assigned to PLCs based on nTotal. The cumulative sum of nTotal was continuously plotted and observed as the concepts were being analyzed (see Fig. 4). Analyzed laboratory concepts that have an nTotal above 50 covered the complete transition and the steepest change of the slope of cumulative total frequencies (Orange graph in Fig. 4). Based on this, we have only included the first 55 analyzed laboratory concepts (PLCs) that have an nTotal above 50.

**Fig. 5:**
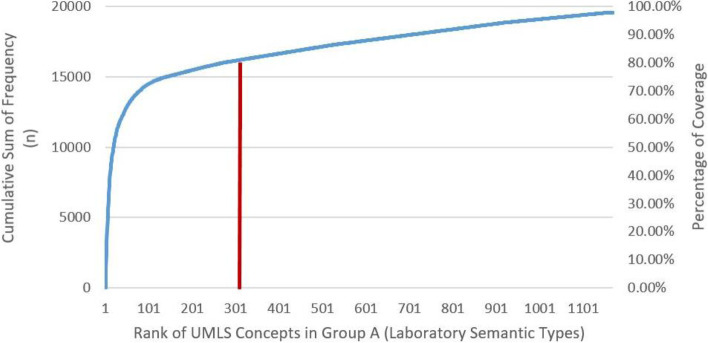
Plot diagram showing the coverage of laboratory concepts within the group of laboratory semantic types (Group A). 311 laboratory concepts representing our 55 LP cover 77.87% of all concept occurrences in group A. n: Frequency of Individual Concept

The final results of the semi-automated analysis included 55 PLCs as well as 648 SLCs and comprised 703 unique UMLS concepts (2.66% of the 26,413 total unique concepts in all included EC forms). Among the 703 unique laboratory concepts included in our final analysis, we identified 311 unique concepts that belong to the group of laboratory semantic types (Group A). These 311 concepts comprised 26.23% of the 1176 concepts in group A and covered 77.87% of its total occurrences (*n* = 15,230/19558). The plot in Fig. 5 shows the cumulative frequency of UMLS concepts in the group of laboratory semantic types (Group A) in terms of simple frequency (n). The complete table of results of the manual analysis of PLCs and SLCs can be found in Appendix 7.

### Top UMLS laboratory concepts in screening eligibility criteria forms

The most frequent UMLS laboratory concept in our analysis of screening EC forms was Measurement of Creatinine in Blood with an nTotal of 1817. Table 1 shows a list of the top 55 UMLS laboratory concepts in screening EC forms.

**Table 1:** Top 55 screening LP ranked according to total frequencies. LP are listed using UMLS and LOINC Terminologies

Rank	1ry LOINC Code	UMLS Lab. Procedure	UMLS Definition	nTotal
1	59,826–8	C3525719	Measurement of creatinine in blood	1817
2	7918–6	C3714540	HIV Antibody Measurement	1670
3	13,955–0	C0201487	Hepatitis C antibody measurement	1595
4	76,625–3	C0201836	Alanine aminotransferase measurement	1464
5	63,557–3	C0201477	Hepatitis B surface antigen measurement	1462
6	2106–3	C0546577	HCG Pregnancy Test	1383
7	26,515–7	C0032181	Platelet Count measurement	1381
8	1920–8	C0201899	Aspartate aminotransferase measurement	1303
9	54,363–7	C0201913	Bilirubin, total measurement	1265
10	4548–4	C0474680	Hemoglobin A1c measurement	1021
11	59,260–0	C0518015	Hemoglobin measurement	968
12	26,511–6	C0948762	Absolute neutrophil count	851
13	2164–2	C0373595	Creatinine clearance measurement	758
14	15,074–8	C0392201	Blood glucose measurement	633
15	69,405–9	C3811844	Estimated Glomerular Filtration Rate	513
16	26,464–8	C0023508	White Blood Cell Count procedure	487
17	19,197–3	C0201544	Prostate specific antigen measurement	471
18	77,145–1	C0428568	Fasting blood glucose measurement	400
19	5010–4	C1533728	Hepatitis C virus genotype determination	352
20	72,383–3	C5189164	HER2 in tissue by immunoassay	324
21	1783–0	C0201850	Alkaline phosphatase measurement	287
22	14,130–9	C3811131	Estrogen Receptor Measurement	283
23	10,676–5	C1868902	HCV viral load	257
24	70,218–3	C0202236	Triglycerides measurement	195
25	6298–4	C0729816	Blood potassium measurement	194
26	34,714–6	C0525032	International Normalized Ratio	191
27	40,557–1	C0373717	Progesterone receptor assay	180
28	1996–8	C0201925	Calcium measurement	171
29	5964–2	C0033707	Prothrombin time assay	137
30	14,913–8	C0853134	blood testosterone measurement	131
31	54,347–0	C0201838	Albumin measurement	130
32	14,647–2	C0201950	Cholesterol measurement test	130
33	22,748–8	C0202117	Low density lipoprotein cholesterol measurement	126
34	69,739–1	C0202274	Urine drug screen	124
35	26,446–5	C2697913	Leukemic Blast Count	122
36	1986–9	C0202100	Insulin C-peptide measurement	118
37	13,954–3	C3835873	Serum Hepatitis B E Antigen, qualitative	113
38	3015–5	C0202230	Thyroid stimulating hormone measurement	111
39	50,564–4	C0042014	Urinalysis	102
40	33,763–4	C1533071	N terminal pro-brain natriuretic peptide level	99
41	82,904–4	C2074589	chromosome studies Philadelphia	95
42	20,570–8	C0018935	Hematocrit procedure	93
43	3173–2	C0030605	Activated Partial Thromboplastin Time measurement	93
44	83,098–4	C0202022	Follicle stimulating hormone measurement	83
45	53,962–7	C0201539	Alpha one fetoprotein measurement	80
46	10,438–0	C3540684	CD20 Expressing Cell Measurement	76
47	76,485–2	C0201657	C-reactive protein measurement	74
48	58,410–2	C0009555	Complete Blood Count	71
49	42,595–9	C3641250	Hepatitis B DNA Measurement	68
50	14,646–4	C0428472	Serum HDL cholesterol measurement	63
51	20,564–1	C0523807	Oxygen saturation measurement	60
52	30,395–8	C0857490	Granulocyte count	58
53	29,760–6	C0201916	Bilirubin, direct measurement	53
54	72,903–8	C0005845	Blood urea nitrogen measurement	51
55	1992–7	C0201924	Calcitonin measurement	50

### Mapping to LOINC and generation of Core dataset

**Fig. 6:**
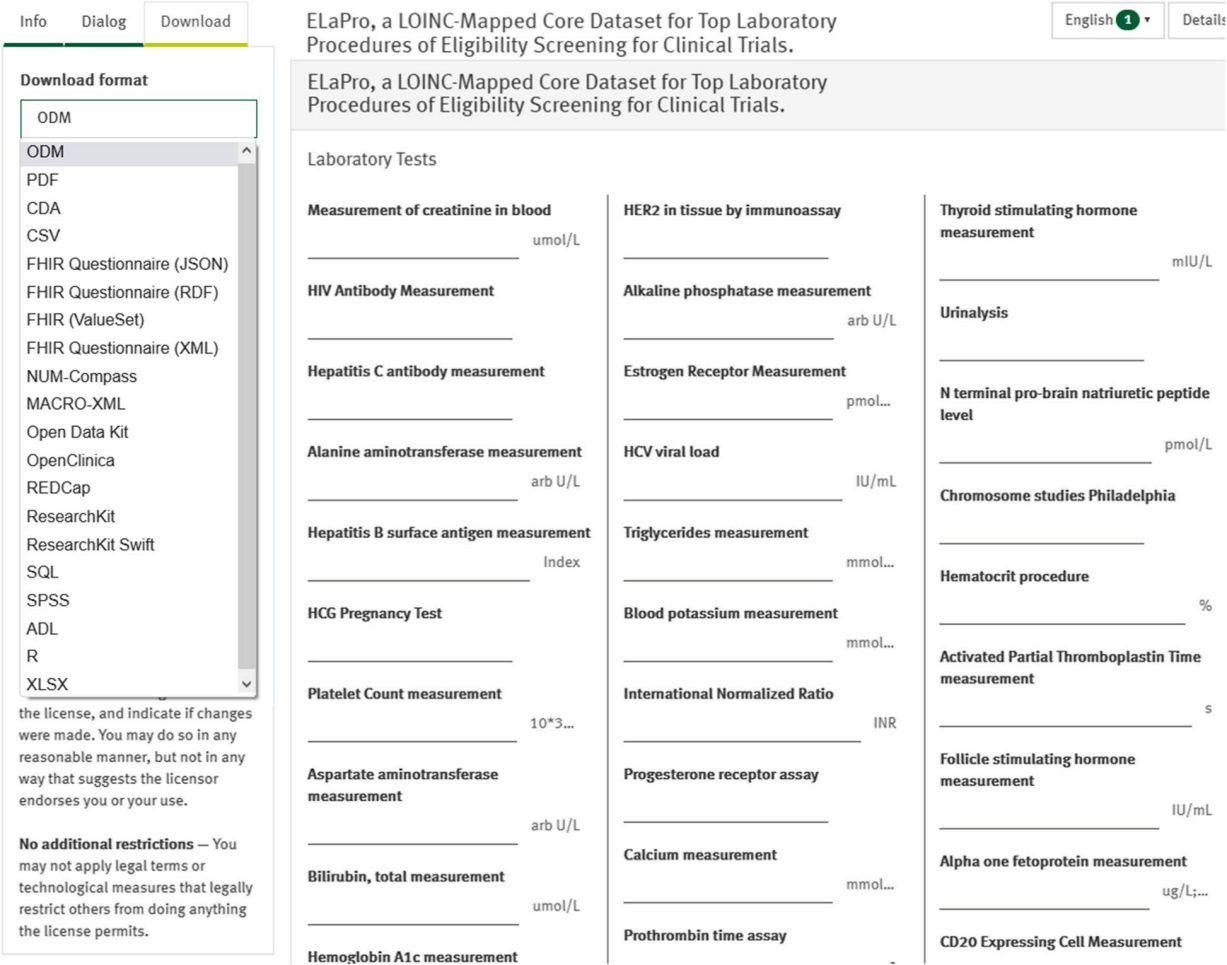
Screenshot of the dataset on MDM portal showing available download formats e.g. ODM and FHIR

The 55 UMLS LP resulted from this analysis were mapped to LOINC terminology as previously explained in the ‘Methods’. Using assigned primary and secondary LOINC concepts, a core dataset was created by completing other LOINC details using LOINC database. The core dataset is available in machine-readable ODM and HL7 FHIR files (see Fig. 6) in UMLS and LOINC terminologies at 10.21961/mdm:44732. CSV, ODM and FHIR formats of the dataset are found in Appendix 8A-8C.

## Discussion

### Principal findings

The purpose of this study is to identify the LP most frequently needed to recruit patients for clinical trials and evaluate the feasibility of establishing a core dataset that can be used by tools of automated screening to help improve patient enrollment in clinical trials. The results show that only a small number of LP is frequently requested in most screening EC, clearly more than other LP, an observation that can be clearly seen in the coverage graph depicted in Figs. 4 and 5 where 311 UMLS concepts representing only 55 LP covered 77.87% of all laboratory concept occurrences in screening EC forms. These findings clearly confirm the feasibility of creating a core dataset.

The results of this analysis include beside the dataset for LP, another dataset for the complete set of UMLS concepts and their 118 semantic types identified in 10,000+ EC forms. These results could further contribute to the improvement of clinical research by serving as a rich source of data for researchers studying complexity and semantic content of EC. They can also be utilized as raw data to perform further analyses on other semantic domains, which might produce new core datasets that could contribute further to the enhancement of automated screening for clinical trials.

### Comparison to earlier data models

Weng et al. extracted a list of the most frequent tags from EC text of 137,889 clinical trials by applying a pure Natural Language Processing (NLP) approach [67]. Weng’s list included 115 general tags in EC and did not focus on a certain domain, his list included only 20 concepts that may directly or indirectly refer to a LP, ELaPro overlapped with 17 of these concepts (10 PLCs and 7 SLCs**).** Doods et al. applied expert-knowledge to manually analyze 17 clinical trials and data elements of EHR systems of several hospitals in Europe to introduce a data inventory of 75 frequent research medical concepts that are available as data items in EHR systems [68]. Dood’s data inventory comprised 41 laboratory concepts in the domain “Laboratory Finding”, 29 of which overlapped with the dataset ELaPro**.** Kury et al. introduced a dataset of most frequent medical concepts of EC from 1000 random clinical trials divided into 8 semantic domains using manual annotation of concepts into a web-based tool followed by an NLP analysis approach [69]. Kury sorted their results into domains, e.g. Device, Condition and Measurement etc. and analyzed the 15 most common concepts of each domain. Laboratory concepts were part of the domain “Measurement” and comprised 9 of its 15 most common tokens. All nine laboratory concepts from Kury’s dataset were part of ELaPro. All these findings provide supporting evidences of the accuracy and generalizability of our results.

While most of these relevant studies presented a general analysis of semantic domains of EC, our study introduces a specialized analysis for one entity of EC that is considered common and optimal for automated queries of EHR systems, i.e., LP, for which a clear gap in research and data models exist [51–53]. ELaPro is the result of analyzing a large number of UMLS-annotated screening EC forms (19516), thereby clearly exceeding the sample size of many relevant earlier studies. Furthermore, the EC forms used in this analysis covered almost all MeSH disease domains (see Fig. 3), which produces more representative results and eliminates the bias that might come from being restricted to a specific clinical domain.

We noticed in our literature review that most of earlier work have utilized NLP methods to provide a general approach to semantic domains of EC with very little to no focus on LP. In 2019 Fraser et al. used 3 pre-trained datasets to study the performance of NLP approaches including “Deep Learning” methods in entity recognition, which is essential when studying fine-grained entities of EC like LP [70]. Several methods performed poor (F1 Score = 0.63) on the largest dataset, “MedMentions”, that contains over 4000 biomedical abstracts, annotated for UMLS semantic types, suggesting potential challenges when solely applying current NLP techniques to real-world data in the absence of a manual expert review [70, 71]. Recent NLP-based systems like Criteria2Query and ElilE achieved relatively better F1 scores in entity recognition (up to 0.795 and 0.79, respectively) [72, 73]. A more laboratory oriented system called Valx showed an F1 score above 0.97, however, this was only tested on a small entity (Diabetes Mellitus I and II). Recent NLP-based systems like Criteria2Query, ElilE or Valx provide a more scalable informatics approach [72–74]. However, our approach puts emphasis on highest accuracy of the results through physician-based curation.

### Strengths

Many earlier studies that analyzed EC were based completely or to a large extent on automated approaches like NLP, whose performance in analyzing fine-grained entities might be suboptimal compared to studies that involve manual expert review to process their data models similar to our study. ELaPro is a novel core dataset, not only because it combines an automated approach of semantic analysis followed by an intensive manual expert review to analyze complex EC patterns and concepts, but also because it represents the first public specialized dataset of LP in eligibility screening combining the UMLS with LOINC, one of most widely-adopted international references for laboratory concepts. The dataset ELaPro provides all potential LOINC-codes to the main element of each of the top LP which allows the user of this dataset to choose the code version that is most commonly used in their health system. ELaPro is also available in interoperable machine-readable formats like Operational Data Model (ODM) and Fast Health Interoperability Resources (FHIR) [75, 76].

### Applications of ELaPro

ELaPro can serve as a data model in automated queries applied to EHR systems to automatically retrieve patients’ electronic data based on their laboratory results meeting certain criteria set by clinical researchers which will optimize patient recruitment for clinical trials. ELaPro can also be useful in enhancing the interface between study feasibility platforms, i.e. planning feasibility of certain clinical research projects by evaluating the ability to find sufficient number of right candidates in a timely manner.

### Limitations and challenges

Approach and Scalability: We realize that our approach does not provide an ultimate solution to automating patient recruitment, mainly due to lacking threshold values and comparable operators. However, providing an expert-curated dataset of the most common laboratory concepts can contribute to automate eligibility screening in many different systems.

The automated part of the analysis facilitates extraction and analysis of UMLS codes from a large number of pre-annotated forms from the MDR of MDM portal. Furthermore, PLC and SLC terms illustrate the importance of manual curation in dealing with the issues of UMLS like redundancy and semantic complexity. While our expert-based approach is not scalable in general, it ensures high accuracy in finding relevant lab concepts in ambiguous text strings of complex EC, which commonly lack entries and links in the LOINC or UMLS terminology system.

Potential Biases: The vast majority of EC analyzed in this study were originally taken from ClinicalTrials.gov, which could pose a potential bias towards trials from the United States of America. Furthermore, the distribution of MeSH disease domains among included forms (Fig. 3) could also pose a potential bias towards the domains that are relatively more represented, i.e. neoplasms, cardiovascular and immune system diseases. Our work aims to study a representative sample of all major MeSH disease entities to produce a general core dataset of common LP. This set can be applied in EHR systems to enhance participant recruitment for clinical studies in many different domains.

The complexity and redundancy of UMLS metathesaurus: Clinical terminologies have a complex semantic structure with redundant or duplicate concepts, this is a known issue in terminologies like UMLS [77], especially when it comes to components of laboratory tests. For instance, the same text string “Albumin” could refer to six semantically different concepts in UMLS (“Biologically Active Substance”, “Amino Acid”, “Peptide, or Protein”, “Gene or Genome”, “Laboratory Procedure”, “Clinical Attribute” or “Physiologic Function”). This issue poses challenges for automatic semantic processing of EC. In addition, many EC use names of pathologic conditions that imply the need to perform a LP rather than directly mentioning the name of the component to be tested, e.g. “Leukocytosis”, which refers to elevated white blood cells in blood belongs to semantic type “Finding”, yet implies the need to perform an LP to fulfill the criteria. Physician-based curations ensure semantic correctness of mapping text strings from EC to clinically relevant laboratory concepts defined in medical terminologies**.** This problem has led to many LP being confusingly annotated by redundant concepts with non-laboratory semantic types. This issue was dealt with in the manual part by using the hierarchy of PLC and SLC, where SLC’s represented all the possible redundant concepts that refer to the main laboratory concept (PLC).

Certain EC concepts like “Leukocytosis”, which do not belong to the two main laboratory semantic types, yet indirectly imply the need for a LP, posed a challenge to this study as they are not amenable to automated semantic analysis. In most instances, this problem is solved by the manual expert review performed by a physician, but in some instances, the EC was vague or ill-defined, in which a LP is implied without specifying the exact component to be tested, e.g. “Abnormal Liver Function”, in this case, these concepts were excluded from the analysis.

### Future directions

Further work is needed to disentangle the above mentioned challenges. Similar approach can be taken to study other semantic domains of EC and produce dedicated in-depth analyses in order to introduce high quality core datasets that help standardize knowledge representation of EC and improve patient recruitment in clinical trials.

## Conclusion

In this study we present ELaPro, the first specialized public core dataset for the most frequent 55 laboratory procedures in EC of clinical trials. This semi-automated study proves the feasibility of establishing such a dataset. The extensive manual expert curation of LP in UMLS-annotated EC and mapping results to the widely-adopted laboratory reference, i.e. LOINC distinguishes the dataset ELaPro from previous work. ELaPro is available in machine-readable formats like CSV, ODM and HL7 FHIR and can serve as a blueprint data model in automated queries applied to EHR systems to optimize patient recruitment in clinical trials and enhance the function of study feasibility platforms. Similar approach could be taken to study other semantic domains of EC and further research should try to solve problems like scalability and redundancy of concepts in complex medical terminologies like UMLS.

## Supplementary Information

**Additional file 1.** Appendix 1: A complete list of names and URIs s of all included EC forms from MDM Portal.

**Additional file 2.** Appendix 2A. A list of unique UMLS concepts of group A sorted by absolute frequency. B: A list of unique UMLS concepts of group B sorted by absolute frequency.

**Additional file 3.** Appendix 3: A complete detailed list of all occurrences of all UMLS concepts identified within EC forms. This database includes details like the question text, names and IDs of all item groups and items as well as UMLS preferred definition of every single UMLS concept occurrence identified within EC forms.

**Additional file 4.** Appendix 4: A schematic illustration of the manual part of the analysis.

**Additional file 5.** Appendix 5: A table of MeSH categories in eligibility criteria forms sorted according to absolute frequencies (n).

**Additional file 6.** Appendix 6: A Table of UMLS semantic types in eligibility criteria forms sorted by absolute frequencies (n).

**Additional file 7.** Appendix 7: A complete list of all UMLS primary and secondary laboratory concepts used in the manual analysis to produce the dataset of top 55 most common Laboratory Procedures. This includes 703 unique UMLS concepts, among which 311 concepts belong to Group A (Concepts of laboratory semantic types). Concepts are sorted in 55 Ranks according to the nTotal of primary and secondary concepts of each Laboratory Procedure.

**Additional file 8.** Appendix 8A: The final dataset, ELaPro, in CSV format after complete mapping to LOINC. B: The final dataset, ELaPro, reported as ODM file (CDASH standard). 8C. The final dataset, ELaPro, in in FHIR format (HL7 standard).

## Data Availability

The eligibility screening forms analyzed in this study are available in the Meta-Data Repository of the Medical-Data-Models Portal https://medical-data-models.org (Category: Eligibility Determination). Generated data are available as downloadable, machine-readable ODM and FHIR files at 10.21961/mdm:44732.

## Abbreviations

ELaPro: Eligibility Laboratory Procedures
EC: Eligibility Criteria
EHR: Electronic Health Record
LP: Laboratory Procedure(s)
UMLS: Unified Medical Language System
MDM: Medical Data Models
LOINC: Logical Observation Identifiers Names and Codes
n: Frequency of Occurrence
STR: String
SAB: Abbreviated Source Name
CUI: Concept Unique Identifier
STY: Semantic Type
NLM: National Library of Medicine
nTotal: Total count of frequencies
MeSH: Medical Subject Headings
PLC: Primary Laboratory Concept
SLC: Secondary Laboratory Concept
ODM: Operational Data Model
FHIR: Fast Health Interoperability Resources
CSV: Comma-Separated Values
