# Supplementary material for: ELaPro, a LOINC-mapped core dataset for top laboratory procedures of eligibility screening for clinical trials
Source: BMC Med Res Methodol. 2022 May 14;22:141. doi: 10.1186/s12874-022-01611-y (PMC9107639; doi:10.1186/s12874-022-01611-y)

Appendix 1: Schematic representation of concepts used in the manual analysis of results. UMLS: Unified Medical Language System; EC: Eligibility Criteria; CUI: Concept Unique Identifier; STR: String; n: Frequency of occurrence for a single concept; STY: Semantic Type; PLC: Primary Laboratory Concept; SLC: Secondary Laboratory Concept; nTotal: Total count of occurrences of all relevant concepts; MII: Medical Informatics Initiative; LOINC: Logical Observation Identifiers Names and Codes; LOINC_NUM: Concept identifier in LOINC terminology.

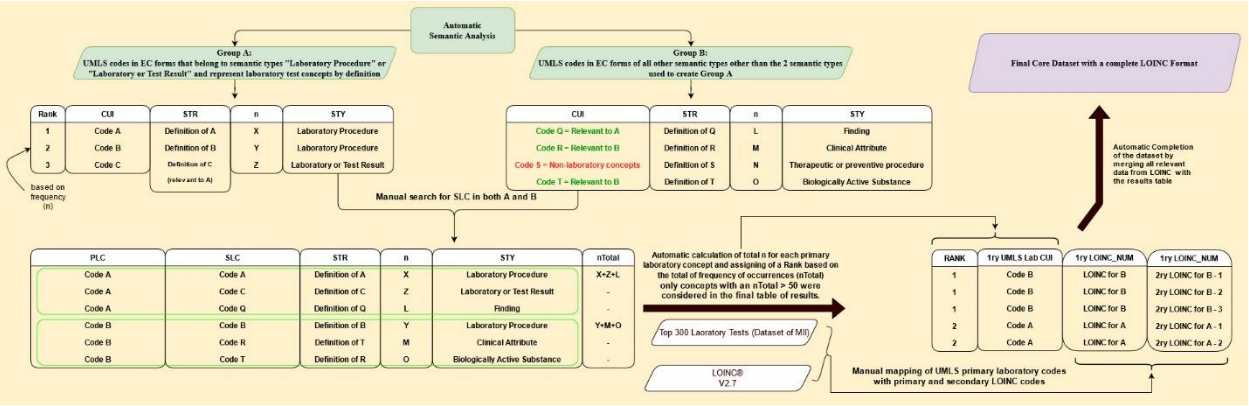

Supplement: Supplementary file 4 — Additional file 4. Appendix 4: A schematic illustration of the manual part of the analysis. [file 12874_2022_1611_MOESM4_ESM.pdf]
