# Supplementary material for: ELaPro, a LOINC-mapped core dataset for top laboratory procedures of eligibility screening for clinical trials
Source: BMC Med Res Methodol. 2022 May 14;22:141. doi: 10.1186/s12874-022-01611-y (PMC9107639; doi:10.1186/s12874-022-01611-y)
Supplement: Supplementary file 5 — Additional file 5. Appendix 5: A table of MeSH categories in eligibility criteria forms sorted according to absolute frequencies (n). [file 12874_2022_1611_MOESM5_ESM.pdf]

Appendix 2: Table of MeSH categories in eligibility criteria forms sorted according to absolute frequencies (n).

No.	MeSH Category	n
1	Neoplasms [C04]	3381
2	Cardiovascular Diseases [C14]	1982
3	Immune System Diseases [C20]	1531
4	Skin and Connective Tissue Diseases [C17]	1436
5	Nutritional and Metabolic Diseases [C18]	1243
6	Endocrine System Diseases [C19]	1191
7	Digestive System Diseases [C06]	954
8	Psychiatry and Psychology [F01-F04]	880
9	Male Urogenital Diseases [C12]	731
10	Hemic and Lymphatic Diseases [C15]	697
11	Nervous System Diseases [C10]	612
12	Musculoskeletal Diseases [C05]	531
13	Pathological Conditions, Signs and Symptoms [C23]	523
14	Respiratory Tract Diseases [C08]	510
15	Infections [C01]	448
16	Female Urogenital Diseases and Pregnancy Complications [C13]	409
17	Otorhinolaryngologic Diseases [C09]	63
18	Stomatognathic Diseases [C07]	60
19	Vaccines [D20.215.894]	45
20	Congenital, Hereditary, and Neonatal Diseases and Abnormalities [C16]	44
21	Eye Diseases [C11]	37
22	Chemically-Induced Disorders [C25]	21
23	Wounds and Injuries [C26]	11
