# Supplementary material for: ELaPro, a LOINC-mapped core dataset for top laboratory procedures of eligibility screening for clinical trials
Source: BMC Med Res Methodol. 2022 May 14;22:141. doi: 10.1186/s12874-022-01611-y (PMC9107639; doi:10.1186/s12874-022-01611-y)
Supplement: Supplementary file 6 — Additional file 6. Appendix 6: A Table of UMLS semantic types in eligibility criteria forms sorted by absolute frequencies (n). [file 12874_2022_1611_MOESM6_ESM.pdf]

Appendix 3: Table of UMLS semantic types in eligibility criteria forms sorted by absolute frequencies (n).

	STY	n
1	Finding	3849
2	Disease or Syndrome	3047
3	Pharmacologic Substance	2201
4	Therapeutic or Preventive Procedure	2140
5	Neoplastic Process	1204
6	Laboratory Procedure	845
7	Diagnostic Procedure	777
8	Qualitative Concept	685
9	Intellectual Product	680
10	Pathologic Function	571
11	Body Part, Organ, or Organ Component	569
12	Functional Concept	492
13	Medical Device	487
14	Health Care Activity	446
15	Organic Chemical	441
16	Mental or Behavioral Dysfunction	439
17	Amino Acid, Peptide, or Protein	425
18	Sign or Symptom	416
19	Quantitative Concept	390
20	Temporal Concept	332
21	Laboratory or Test Result	331
22	Spatial Concept	319
23	Clinical Attribute	283
24	Injury or Poisoning	246
25	Idea or Concept	183
26	Manufactured Object	168
27	Biologically Active Substance	162
28	Population Group	159
29	Food	140
30	Research Activity	137
31	Organ or Tissue Function	130
32	Body Location or Region	129
33	Health Care Related Organization	129
34	Immunologic Factor	128
35	Gene or Genome	121
36	Professional or Occupational Group	117
37	Cell or Molecular Dysfunction	114
38	Individual Behavior	109
39	Organism Function	108
40	Molecular Function	106
41	Mental Process	102
42	Cell	101
43	Indicator, Reagent, or Diagnostic Aid	94
44	Clinical Drug	90
45	Anatomical Abnormality	89
46	Congenital Abnormality	88
47	Activity	82
48	Biomedical or Dental Material	82
49	Classification	81
50	Organism Attribute	79
51	Body Space or Junction	78
52	Body Substance	72
53	Physiologic Function	72
54	Geographic Area	71
55	Biomedical Occupation or Discipline	67
56	Conceptual Entity	65
57	Tissue	62
58	Antibiotic	59
59	Daily or Recreational Activity	57
60	Hormone	55
61	Social Behavior	55
62	Natural Phenomenon or Process	48
63	Receptor	47
64	Family Group	45
65	Patient or Disabled Group	45
66	Plant	45
67	Nucleic Acid, Nucleoside, or Nucleotide	41
68	Body System	40
69	Virus	38
70	Acquired Abnormality	37
71	Inorganic Chemical	37
72	Genetic Function	34
73	Phenomenon or Process	33
74	Substance	33
75	Bacterium	31
76	Cell Function	28
77	Occupational Activity	27
78	Element, Ion, or Isotope	25
79	Language	25
80	Educational Activity	24
81	Regulation or Law	21
82	Cell Component	20
83	Governmental or Regulatory Activity	20

**84**	Age Group	19
**85**	Anatomical Structure	19
**86**	Chemical Viewed Functionally	19
**87**	Organization	19
**88**	Biologic Function	18
**89**	Hazardous or Poisonous Substance	18
**90**	Molecular Biology Research Technique	18
**91**	Occupation or Discipline	17
**92**	Chemical Viewed Structurally	13
**93**	Fungus	11
**94**	Mammal	11
**95**	Eukaryote	10
**96**	Event	10
**97**	Group Attribute	9
**98**	Machine Activity	9
**99**	Professional Society	9
**100**	Vitamin	9
**101**	Group	8
**102**	Behavior	7
**103**	Enzyme	7
**104**	Human-caused Phenomenon or Process	7
**105**	Nucleotide Sequence	7
**106**	Chemical	6
**107**	Organism	6
**108**	Physical Object	5
**109**	Embryonic Structure	4
**110**	Self-help or Relief Organization	4
**111**	Entity	3
**112**	Research Device	3
**113**	Bird	2
**114**	Human	2
**115**	Animal	1
**116**	Drug Delivery Device	1
**117**	Environmental Effect of Humans	1
**118**	Fish	1
